# Attitudes of health professionals towards suicidal behavior: an intervention study

**DOI:** 10.11606/s1518-8787.2022056003320

**Published:** 2022-06-07

**Authors:** Jesiele Spindler Faria, Samira Reschetti Marcon, Alice Milani Nespollo, Hugo Gedeon Barros dos Santos, Mariano Martínez Espinosa, Kleici Kleslly Brito de Oliveira, Josemara Gomes da Silva Lima

**Affiliations:** I Universidade Federal de Mato Grosso Faculdade de Enfermagem. Cuiabá MT Brasil Universidade Federal de Mato Grosso. Faculdade de Enfermagem. Cuiabá, MT, Brasil; II Universidade Federal de Mato Grosso Faculdade de Enfermagem Sinop MT Brasil Universidade Federal de Mato Grosso. Faculdade de Enfermagem - Campus Sinop. Sinop, MT, Brasil; III Hospital Universitário Júlio Muller Unidade de Atenção Psicossocial Cuiabá MT Brasil Hospital Universitário Júlio Muller. Unidade de Atenção Psicossocial. Cuiabá, MT, Brasil; IV Universidade Federal de Mato Grosso Instituto de Ciências Exatas e da Terra Departamento de Estatística Cuiabá Mato Grosso Brasil Universidade Federal de Mato Grosso. Instituto de Ciências Exatas e da Terra. Departamento de Estatística. Cuiabá, Mato Grosso, Brasil; V Universidade Federal de Mato Grosso Programa de Pós-Graduação em Saúde Coletiva Cuiabá MT Brasil Universidade Federal de Mato Grosso. Programa de Pós-Graduação em Saúde Coletiva. Cuiabá, MT, Brasil

**Keywords:** Attitude of Health Personnel, Health Human Resource Training, Suicidal Ideation, Primary Health Care, Randomized Controlled Trial

## Abstract

**OBJECTIVE:**

To evaluate the effect of an educational intervention on the attitudes of primary healthcare providers regarding patients with suicidal behavior.

**METHODS:**

Clinical trial randomized by clusters, with a sample of 261 healthcare professionals, from 22 health units selected by stratified sampling, were chosen and randomly allocated, by drawing, into two groups: intervention (n = 87) and control (n = 174). The participants of the intervention group were exposed to a 20-hour training on suicidal behavior. All 261 participants were evaluated before and after the intervention; the groups were compared to evaluate their attitude towards suicidal behavior using the Suicide Behavior Attitude Questionnaire (SBAQ), an evaluation made by comparison of the means via t-Student test, for independent samples, and paired t-test, for dependent samples.

**RESULTS:**

The intervention group, in comparison to their evaluation before and after training, as well as in the comparison with the evaluation of the control group, showed statistically significant differences in attitudes towards suicidal behavior, according to the differences presented in the scores for the domains: “perception of professional capacity,” in all four items; “negative feeling,” in six of the seven items; and in the “right to commit suicide” domain, in three of the five items.

**CONCLUSION:**

The brief training developed in primary health care was effective to improve the attitudes of the participants who were part of the intervention group regarding patients with suicidal behavior.

## INTRODUCTION

Approximately 800,000 people commit suicide annually, which corresponds to a rate of 11.4 deaths per 100,000 inhabitants worldwide, being among the top ten death causes in all age groups^1^. Although the suicide mortality rate in some Western European countries has decreased in recent decades, other countries, such as Mexico, the United States, and Brazil, experienced an increase in cases during the same period^2^. According to national data, more than 10,000 Brazilians committed suicide, meaning 5.5 occurrences per 100,000 inhabitants, in 2015, and 6.5 deaths per 100,000 inhabitants, in 2016, proving an increase over the years^3^. Considering the magnitude of the problem, the World Health Organization – WHO recommends that the prioritization of suicide, both in the care offered and in the development of public health policies^1^.

In this context, the mobilization of the health services is inevitable, since the primary care environment often provides the initial actions for mental health care^4^. This is directly related to the insufficient number of professionals in the psychosocial care network to meet this demand, especially in middle-income countries, such as Brazil, where the coverage of these services encompass only 10% of the individuals who need it^4^.

A systematic review analyzed the contact individuals had with primary healthcare providers before committing suicide and the results showed that, on average, 80% sought the health service in the year prior to suicide and 44%, in the same month of death^5^. Similar findings were described in a Norwegian study that investigated the use of primary healthcare services in 4,926 suicide cases (subjects aged > 15 years) from 2007 to 2015. The results showed that approximately 90% of the individuals consulted a generalist professional in primary care in the year prior to suicide and up to 46.4% in their last month of life^6^.

This suggests that professionals of this level of care are in a unique position to identify and intervene in subjects at suicidal risk, since primary health care is the first contact with the health network^1^. Most professionals, however, manifest negative attitudes when dealing with people with suicidal behavior^7^, commonly due to factors such as unpreparedness or difficulties in dealing with this demand, providing limited initial care, and often referring patients to other services^9,10^, compromising the quality of care provided.

An attitude can be defined as a set of cognitive, affective, and behavioral attributes. Therefore, attitude is an inclination of the individual – acquired socially, from personal experiences, and from personality factors – to act in a specific way in relation to certain people, objects, and situations^11^.

In a meta-analysis that reviewed studies developed until 2018, negative attitudes, limited empathy, and some levels of hostility were observed from healthcare providers when attending to people with suicidal behavior. The data also indicated that training and professional qualification on how to deal with these cases promote more positive attitudes^12^. Corroborating these findings, subsequent studies observed a resistance in attending this clientele; care based on beliefs and stigmas; technical and routine activities prioritized over psychological support; deficient knowledge and skills; in addition to the need for training to facilitate therapeutic relationships^13^.

Studies that propose to analyze the effects of educational strategies on the modification of attitudes, although scarce in the Brazilian and international literature, show a predominance in interventions that have the hospital environment as the targeted audience^16^.

Thus, this study questions: can the attitudes of primary healthcare providers be modified after professional training on dealing with suicidal behavior? To answer this question, our study aims to evaluate the effect of an educational intervention on the attitudes of primary healthcare providers regarding suicidal behavior.

## METHOD

This is a two-arm parallel randomized controlled trial inscribed in the Brazilian Registry of Clinical Trials (ReBEC) under the code RBR-9pmjf5w.

Developed in the city of Cuiabá, from May to August 2017, in Family Health Strategy (FHS) units, structured by multidisciplinary teams, composed of at least one nurse, a general practitioner or family doctor, a nursing technician, and community health agents, and can also have an expanded team, including oral health professionals. The city of Cuiabá, in the state of Mato Grosso, has 70 FHS teams, 3 in the rural area of the municipality and 67 in the urban area, distributed in four regions: 24 units in the northern region, 21 in the southern, 11 in the eastern, and 11 in the western. Totaling 298 healthcare providers, including physicians, nurses, and nursing technicians; 697 community health agents; and 86 oral health professionals^23^.

The study population consisted of healthcare providers who functioned as physician, nurses, and nursing technicians; excluding from the study professionals from teams located in the rural area (since they composed the pilot test sample of this study), as well as health agents and oral health professionals (since the instrument used does not target these categories).

A probabilistic sampling was obtained by clustering and stratification; the sampling unit was the health team, composed of a group of professionals, which was stratified by regional health units.

To determine the number of subjects, a formula was used for paired data^24^, in which the mean of the changes in attitude (pre- and post-intervention) and the standard deviations, obtained from a reference study, were determined^25^. A standard deviation of 2.92 was estimated, considering a minimum difference to be detected of 1.0, 95% confidence, and power of 0.80. Thus, the initial sample size adjusted for population (n = 298) was 56 individuals. Since it is a cluster sampling design, a design effect factor was established at 1.22 and an estimated non-response rate was estimated at 22%, which resulted in a final sample of 87 individuals for the intervention group. For the control group, 1 for 2 was considered, i.e., 174 individuals.

The random selection of participants for the intervention and control groups was performed by cluster sampling and stratification proportional to the size of the population, considering the regions (north, south, east, west) as strata and the FHS teams as randomization units, in order to obtain adequate control and reduce methodological bias. The number of professionals selected in each region was defined by multiplying the fraction of professionals by the sample size (87). Subsequently, to define the number of teams to be drawn, the number of healthcare providers per stratum was divided by the number of teams (four), totaling 22 teams randomly drawn by statistical program (Table 1).

**Table 1:** Strata, total FHS teams, number of professionals by region, professionals per team, selected teams, and total participating professionals. Cuiabá, state of Mato Grosso, Brazil, 2017.

Stratum	Number of FHS teams	Total number of Professionals	%	Professionals by team	Number of teams selected	Professionals selected by region
Northern Region	24	115	38.59	4	8	26
Southern Region	21	86	27.85	4	7	19
Eastern Region	11	51	17.11	4	4	12
Western Region	11	49	16.44	4	4	11
**Total**	**67**	**298**	**100**	**4**	**22**	**68**

FHS: family health strategy.

The Suicide Behavior Attitude Questionnaire (SBAQ), composed of clinical situations frequently experienced by healthcare providers, was used to identify the professional’s attitude towards suicidal behavior^26^. This instrument is divided into factors relating to the feelings professionals have in relation to the patient with suicidal behavior, their self-perception on capacity for care, and the right to suicide. Investigating these factors allows for the positive or negative attitudes to be measured. According to the mean obtained by adding the values of each question and diving by the total number of questions in each domain, we can verify a more positive attitudes by the higher scores for the domain “Perception of professional capacity” and by the lower scores for the domains “Feelings towards the patient” and “Right to suicide^26^.” To verify other variables, such as sociodemographic (age, gender, color, education), professional background (occupation, specializations, training in mental health), and professional practice (previous care to patients with suicidal behavior); a closed instrument was constructed, applied with the SBAQ.

At first, all participating units were visited in order to inform about the study and apply the instruments. The professionals selected for the intervention group, were invited to participate in the intervention (training) in addition to answering the instruments (pre-test). The questionnaires were previously coded and allowed pairing, it did not allow, however, for the personal identification of the participants. The participants who were absent from the health unit at the first attempt were sought two more times, at different moments. Whenever possible and necessary, visits were scheduled.

Subsequently, the intervention group was offered a 20-hour training, aiming to improve the ability to recognize the degree of risk of an individual with suicidal behavior and to intervene; to learn strategies to care for and/or to refer individuals with suicidal behavior; and to recognize and improve one’s own attitude towards a patient with suicidal behavior. The content was defined according to the manual of suicide prevention aimed at primary healthcare teams of the World Health Organization. The training was conducted by psychologists and researchers in Suicidology, with extensive clinical and pedagogical experience in this topic. Immediately after the end of the training, the SBAQ was reapplied to the intervention group (post-test). For the control group, no type of intervention was offered, and the post-test was reapplied later in the health units.

The expected primary outcome was a difference in the level of attitudes towards suicidal behavior between the beginning and end of the intervention, verified through the SBAQ, based on the increase of scores equal to or greater than 3%; the analysis was performed on the principle of intention to treat.

Figure shows the flowchart of participation of individuals involved in the clinical trial, from recruitment to the last evaluation. Of the 87 professionals who were initially exposed to the intervention, 69 (79.3%) participated in the last evaluation, and in the control group 88 (50.6%) remained for analysis. The losses in the study occurred due to refusal to remain in the study (n of the intervention group = 7, n of the control group = 11), incomplete filling of the instruments (n of the intervention group = 11, n of the control group = 37), change of workplace or were not found (n of the control group = 38).

**Figure:**
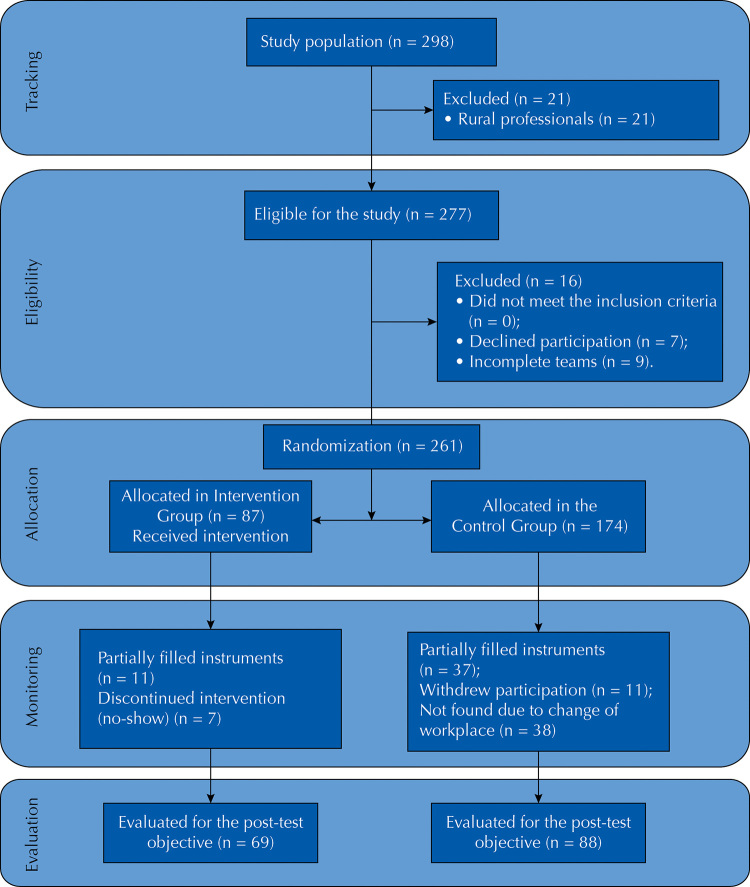
Study flowchart. Cuiabá, state of Mato Grosso, Brazil, 2017.

The distribution of the data was verified by the Shapiro-Wilk test. To compare the two groups, Pearson’s chi-square homogeneity test, t-student test for two independent samples, and U-Mann Whitney test were used. Comparison within the pre- and post-intervention group were performed by the paired t-test for dependent samples and by the Wilcoxon signed-rank test. The level of significance adopted was 5% for all tests. The research project was approved by the research ethics committee in accordance with Resolution 466/2012.

## RESULTS

The analysis of sociodemographic data, of previous training, and of attending to suicidal behavior (Table 2) showed no statistically significant difference between the groups.

**Table 2:** Sociodemographic, professional, and suicidal behavior characteristics of health professionals in the FHS units of Cuiabá, state of Mato Grosso, Brazil, 2017.

Characteristics	Intervention Group	Control group	n (88)	(%)
	n (69)	(%)		
Age group				
20–39	31	44.9	31	35.2
40–59	37	53.6	49	55.7
60–79	1	1.4	8	9.1
Gender				
Woman	60	87.0	79	89.8
Man	9	13.0	9	10.2
Education Level				
Without higher education	11	15.9	15	17.0
With higher education	58	84.1	73	83.0
Marital status				
With a partner (married, stable union)	36	52.2	57	64.8
Without a partner (single, separated, widower)	33	47.8	31	35.2
Religion				
Yes	58	84.1	79	89.8
No	11	15.9	9	10.2
Occupation				
Physician	17	24.6	25	28.4
Nurse	18	26.1	24	27.3
Nursing technician	34	49.3	39	44.3
Mental health training				
Yes	27	39.1	25	28.4
No	42	60.9	63	71.6
Suicide training				
Yes	8	11.6	22	25.0
No	61	88.4	66	75.0
Attended suicidal ideation				
Yes	51	73.9	43	48.9
No	18	26.1	45	51.1
Attended suicide attempts				
Yes	37	53.6	45	51.1
No	32	46.4	43	48.9
Attended patients who have committed suicide				
Yes	18	26.1	20	22.7
No	51	73.9	68	77.3
Have you ever thought of committing suicide				
Yes	11	15.9	11	12.5
No	58	84.1	77	87.5
Have you ever attempted suicide				
Yes	2	2.9	9	10.2
No	67	97.1	79	89.8
In your family has anyone attempted suicide				
Yes	27	39.1	20	22.7
No	42	60.9	68	77.3
In your family has anyone committed suicide				
Yes	12	17.4	9	10.2
No	57	82.6	79	89.8
Among your friends, has anyone attempted suicide				
Yes	27	39.1	21	23.9
No	42	60.9	67	76.1
Among your friends, has anyone committed suicide				
Yes	23	33.3	23	26.1
No	46	66.7	65	73.9

FHS: family health strategy.

Table 3 describes the attitudes of healthcare providers in the intervention group and the control group, pre- and post-test. The results did not reveal significant difference for most items of the SBAQ, between the groups in the pre-test, except for item Q17, which presented statistical significance with ranks mean (intervention group = 1.40; control group = 2.70; p-value < 0.001). However, in the evaluation performed after training, there were statistically significant differences between the two groups in all items of the factors “negative feelings towards the patient” and “perception of professional capacity.” In “right to suicide” of the five items that make up the factor, items Q4, Q6, and Q16 underwent statistically significant changes (p-value < 0.05), however, questions Q3 and Q18 did not present significant changes (p-value = 0.131 and p-value = 0.597, respectively).

**Table 3:** Attitudes of the health professionals of the intervention group and of the control group, before and after training. Cuiabá, state of Mato Grosso, Brazil, 2017.

Factors	Intervention Group pre-test^a^	Control group pre-test^b^	p-value^c^	Intervention Group post-test^d^	Control group post-test^e^	p-value^f^
**Factor 1. Feelings towards the patient**						
Those that keep threating to kill themselves usually do not go through with it.	3.70	4.55	0.020	0.10	4.35	< 0.01
Deep down, I would rather not get too involved with patients who attempted suicide.	1.00	2.85	0.132	0.10	2.00	< 0.01
I am afraid to ask about suicide ideas and end up inducing the patient into it.	1.70	3.00	0.337	1.00	2.55	< 0.01
Deep down, sometimes it is even infuriating, because there is so many people who want to live and that patient wants to die.	1.70	3.50	0.015	0.40	3.80	< 0.001
One feels powerless in front of a person who wants to kill themselves.	5.20	5.70	0.524	2.20	5.30	< 0.001
In the case of patients who are suffering greatly from a physical illness, I find the idea of suicide more acceptable.	1.40	2.70	< 0.01	0.60	2.80	< 0.01
If you really want to kill yourself, you do not keep trying to kill yourself.	1.80	1.85	0.376	0.10	2.00	< 0.01
**Factor 2. Perception of professional capacity**						
I feel like I can help someone who attempted suicide.	5.30	5.35	0.914	8.25	5.85	< 0.01
I feel I am capable to recognize when a patient is at risk of committing suicide.	4.61	4.31	0.479	8.65	3.70	< 0.01
I think I have professional preparation to deal with patients at risk of suicide.	2.50	3.10	0.602	7.50	3.00	< 0.01
I feel insecure to attend patients at risk of suicide.	5.60	6.15	0.614	3.00	6.45	< 0.01
**Factor 3. Right to suicide**						
After all, I think a person has the right to commit suicide.	0.60	1.00	0.618	1.50	1.00	0.131
In the face of a suicide, I think: if someone had talked, the person would have found another way.	8.20	8.10	0.934	9.50	8.00	< 0.01
Life is a gift from God and only He can take away.	9.50	10.00	0.009	8.00	9.50	0.001
Whoever has God in their heart will not try to kill themselves.	4.60	5.30	0.442	1.60	5.15	0.025
When a person talks about ending their life, I try to get that out of their head.	8.90	8.35	0.132	9.00	8.80	0.597

^a^ Intervention Group evaluation prior to the intervention.

^b^ Control Group evaluation prior to the intervention.

^c^ significant p-value less than 0.05 obtained by student’s t-test.

^d^ Intervention group post-evaluation.

^e^ Control group post-evaluation.

^f^ significant p-value less than 0.05 obtained by the U-Mann Whitney test.

Table 4 describes the attitudes of healthcare providers in the intervention group and the control group, pre- and post-test. In the factor “negative feelings in relation to the patient,” we observed statistically significant gains in six of the seven items that make up the domain (Q2, Q5, Q9, Q13, Q15, and Q19). In question 17, the means of the pre-test, 2.71, and post-test, 2.02 (p-value = 0.070), showed differences, but without statistical significance. Regarding the “perception of professional capacity” there were statistically significant changes for all four items of this factor, with p-value < 0.001. In “right to suicide,” of the five items that make up the factor, items Q4, Q6, and Q16 underwent statistically significant changes (p-value < 0.005), however, questions Q3 and Q18 did not present significant changes (p-value = 0.086 and p-value = 0.234, respectively).

**Table 4:** Attitudes of health professionals in the intervention group, before and after the professional training of the FHS in the municipality of Cuiabá, MT, Brazil, 2017.

Factors	Intervention Group pre-test^a^	Group intervention post-test^b^	Z^c^	p^d^
**Factor 1. Feelings towards the patient**				
Those that keep threating to kill themselves usually do not.	3.56	1.12	-5.763	< 0.01
Deep down, I’d rather not get too involved with patients who attempted suicide.	2.13	0.96	-2.778	0.005
I am afraid to ask about suicide ideas and end up inducing the patient into it.	2.90	1.08	-4.244	< 0.01
Deep down, sometimes it is even infuriating, because there is so many people who want to live and that patient wants to die.	3.39	1.26	-4.787	< 0.01
One feels powerless in front of a person who wants to kill themselves.	5.29	3.06	-4.540	< 0.01
In the case of patients who are suffering greatly from a physical illness, I find the idea of suicide more acceptable.	2.71	2.02	-1.814	0.070
If you really want to kill yourself, you do not keep trying to kill yourself.	3.12	0.96	-5.462	< 0.01
**Factor 2. Perception of professional capacity**				
I feel like I can help someone who attempted suicide.	5.38	8.28	-6.323	< 0.01
I feel I am capable to recognize when a patient is at risk of committing suicide.	4.61	7.78	-6.138	< 0.01
I think I have professional preparation to deal with patients at risk of suicide.	3.53	6.81	-5.606	< 0.01
I feel insecure to attend patients at risk of suicide.	5.70	3.55	-4.206	< 0.01
**Factor 3. Right to suicide**				
After all, I think a person has the right to commit suicide.	1.90	2.58	-1.719	0.086
In the face of a suicide, I think: if someone had talked, the person would have found another way.	10.0	7.93	-3.580	< 0.01
Life is a gift from God, and only He can take away.	8.74	7.04	-4.399	< 0.01
Whoever has God in their heart will not try to kill themselves.	4.50	3.25	-3.278	0.001
When a person talks about ending their life, I try to get that out of their head.	8.27	9.46	-1.189	0.234

FHS: family health strategy.

^a^ Intervention Group evaluation before the training.

^b^ Intervention group evaluation after the training.

^c^ Wilcoxon test for paired samples.

^d^ p-value considered significant less than 0.05.

## DISCUSSION

The literature shows that the care given to patients with suicidal behavior can be influenced by several factors, among which are the attitudes of healthcare providers^9,10,12^.

Attending to patients with suicidal behavior can lead to feelings of frustration, impotence, guilt, contempt, and anger^18,27^. Additionally, suicide behavior is surrounded by myths and beliefs, such as those that classify suicide attempts and threats as forms of seeking attention and not as actual intention, or even that people with this behavior are considered cowards^9,30,31^. An attitude is subject to change, which can be evidenced in our study; regarding the “feeling in relation to the patient,” the post-intervention evaluation showed changes for all items in this domain. These findings demonstrated changes in the understanding of suicidal attempts and threats as potential risk factors and that people in these conditions are in intense suffering, providing a more empathic postures in relation to these patients.

The fact that the professional reacts negatively when attending to a patient with suicidal behavior may, among other aspects, be related to the training process that often does not provide tools for coping with situations of death, especially when associated with the subject’s choice^31^. The lack of desire to live can be conflicting for professionals, since they are instructed to save lives. Thus, aspects such as overcoming the dilemmas of omnipotence and omniscience, which usually cause distress in these situations, were widely discussed in training.

Another aspect that was raised is that the feeling of anxiety, due to a possible error of conduct or evaluation, and the idea of responsibility over the patient’s life can be expressed by the difficulty in establishing a bond or in the fear of talking about suicide for fear of inducing the patient to commit it^32^. However, the discussions generated during the training showed that the professionals were less elusive in getting involved and establishing bonds with patients who attempted suicide and presented a reduction in the feeling of impotence. This may explain the significant changes in the questionnaire responses in this factor.

Perceiving one’s own negative feelings as defense mechanisms, considering death as part of human existence, and identifying the feelings involved in the process of death and dying were a part of the methodology employed and favored the understanding and modification of feelings among professionals. These findings corroborate the study developed with healthcare providers in which, after training, a significant reduction in negative feelings and better accuracy in risk assessment were found^19^.

On the “perception of professional capacity,” the findings showed self-perception of greater capacity for care, better professional preparation, and confidence to deal with patients at risk of suicide. Similar results are found in a Japanese multicenter study that performed a two-hour intervention with 74 healthcare providers. There were significant increases in perceived skills, confidence, and attitude, as well as a greater competence in the assessment of suicidal risk, and more confidence in attending to patients in these conditions^33^.

A study conducted in Australia, in which 248 health professionals participated in a training on suicidal behavior, resulted in improved knowledge, more appropriate attitudes regarding expansion of communication capacity, and increased confidence in providing appropriate care^21^. The literature has emphasized that negative attitudes towards suicidal patients among healthcare providers may be more related to lack of knowledge and uncertainties in how to care for than to a specific hostility^20^. Thus, misinformation about suicide can perpetuate a mistaken approach.

Continuously updated knowledge, especially regarding the assessment of suicidal risk and treatment options, can decrease anxiety about failures and increase the perception of professional capacity. As professionals understand and fulfill their responsibilities in identifying, evaluating, and intervening therapeutically, performing professionally according to evidence, and planning the follow-up of a person at suicidal risk, they become aware that the factors related to professional skills and competences have been contemplated^17^.

Healthcare providers live alongside suffering, pain, fear, hopelessness, losses in various ways and often face the processes of death and dying. Feeling helpless and powerless is common in these situations. The belief that only cure or recovery characterizes as good care, emphasized during academic training and reiterated daily by the culture of therapeutic obstinacy, may contribute to professional insecurity when faced with situations that signal a possible self-inflicted death^34^.

Several authors also highlight the influence of organizational issues of the service, as well as the scarcity of physical structure and especially trained personnel, demonstrating the difficulties and fears of not having resources in the face of the unexpected. During the training, several factors such as those reported above were described as limiting an effective care, accompanied by feelings of insecurity, helplessness, and incompetence. It was emphasized, however, that the fear of error can be gradually replaced by the confidence to intervene when limits and potentials are recognized^17,21,35^.

The third component investigated in our study refers to the “right to suicide,” in which we observed changes in three of its five items, in the evaluation after intervention. However, one of the items in which no significant changes were observed was the statement: “despite everything, I think a person has the right to suicide.” An Australian study described similar result after a one-day training: in which only 30% agreed with the right to commit suicide. According to the authors, these findings reflect the nature of the items that make up this factor (moral and religious beliefs) are deeply ingrained, with less expectation of modifications, even after an educational intervention^21^.

A healthcare providers should not prioritize their individual principles and convictions when attending a person with suicidal behavior, with the risk of inducing them to adapt to social standards, based on their personal values and beliefs that may not be relevant to the patient, endangering the therapeutic bond necessary for care. Thus, the care provided cannot, in any way, intensify the feeling of guilt in the person with suicidal ideation or tendency and in their families^34^.

When asked about the possibility of changing a suicide intention by means of a conversation, significant positive effects were perceived after training. However, in the statement “when a person talks about ending their life, I try to get it out of their head,” which assumes that the professional themselves are actively involved in the event, the results were not statistically significant. A possible explanation for this refers to the fact that, although the professional believes in the possibility of prevention using therapeutic communication, they do not recognize themselves as an essential element in this process.

Dealing with death triggers countless reactions in humans, among them the perception of finitude itself. Focusing on exclusively technical, bureaucratic, and routine issues when it comes to this theme is part of a posture of denying death, to the extent that it provides power to the healthcare provider and softens the feeling of impotence^35^. Focusing exclusively on the biological aspects and investing in technological resources as alternatives for prolonging life avoid, to some extent, not only contact with death but also a therapeutic communication that could give access to the patient’s feelings. Thus, indirectly, healthcare providers avoid contact with their own death and with their own emotions^34^.

Refusing to speak or think about death is, in a way, comforting since it feeds a fantasy that death can be driven away by not manifesting it with either words or thoughts. The death of the other is characterized as an announcement and anticipation of one’s own death – a threat – and suicide also translates into a mutilation within society by breaking its natural course, stirring the moral bases^35^.

Suicide, since it involves biological, cultural, and social aspects, demands that educational interventions comprise, in addition to clinical management, an understanding of the psychological distress factors that are involved, which are key elements for the therapeutic approach of the multidisciplinary team. Moreover, it is necessary to rethink suicide prevention strategies to provide knowledge that leads to a gaze less regulated by judgments and moral rules, so that the person who experiences this suffering can be welcomed in any context, allowing better conditions for recovery and social rehabilitation^31^.

This study presents as limitation the difficulty of comparative analyses with other interventions, both because they are scarce in the literature and because they differ substantially in relation to the target audience, content taught, teaching-learning methodologies, or forms of result analysis. However, our contributions lie in the type of experimental design that, by raising awareness to the role of the health professional and to the improvement of knowledge about suicidal behavior, has promoted positive change in attitudes and allowed the perception of capacity and confidence to increase regarding the care for these patients, as suggested by the results.

More positive attitudes were observed among professionals towards suicidal behavior after the intervention. This finding contributes to the quality of care in primary care and reinforces the feasibility of training to prevent this condition in the population. Thus, the data evidenced are relevant both for scientific production and for the reality of the services within in the Brazilian Unified Health System.

The results found in this study are in line with the existing literature and collaborate with the current scientific panorama, as they provide support for the development of strategies that contribute to the reduction of the high rates of attempts and suicides in the country.

We suggest for new studies to conduct sequential evaluation or follow-up, observing whether such changes are sustained along the timeline; in addition to verifying the duration of educational intervention programs, aiming to optimize the time spent in such programs, as well as the contents addressed for validation of standardized educational material in order to test and replicate such results in other populations.
