# Supplementary material for: Attitudes of health professionals towards suicidal behavior: an intervention study
Source: Rev Saude Publica. 2022 Jun 7;56:54. doi: 10.11606/s1518-8787.2022056003320 (PMC9239338; doi:10.11606/s1518-8787.2022056003320)
Supplement: Translation

# Translation: Atitudes dos profissionais de saúde frente a comportamento suicida: estudo de intervenção

**Authors:** Jesiele Spindler Faria, Samira Reschetti Marcon, Alice Milani Nespollo, Hugo Gedeon Barros dos Santos, Mariano Martínez Espinosa, Kleici Kleslly Brito de Oliveira, Josemara Gomes da Silva Lima

**Affiliations:** I Brasil Universidade Federal de Mato Grosso. Faculdade de Enfermagem. Cuiabá, MT, Brasil; II Brasil Universidade Federal de Mato Grosso. Faculdade de Enfermagem - Campus Sinop. Sinop, MT, Brasil; III Brasil Hospital Universitário Júlio Muller. Unidade de Atenção Psicossocial. Cuiabá, MT, Brasil; IV Brasil Universidade Federal de Mato Grosso. Instituto de Ciências Exatas e da Terra. Departamento de Estatística. Cuiabá, Mato Grosso, Brasil; V Brasil Universidade Federal de Mato Grosso. Programa de Pós-Graduação em Saúde Coletiva. Cuiabá, MT, Brasil

**Keywords:** Atitude do Pessoal de Saúde, Capacitação de Recursos Humanos em Saúde, Ideação Suicida, Atenção Primária à Saúde, Ensaio Clínico Controlado Aleatório

## Abstract

**OBJETIVO:**

Avaliar o efeito de uma intervenção educativa sobre comportamento suicida nas atitudes dos profissionais de saúde da atenção primária.

**MÉTODO:**

Ensaio clínico randomizado por conglomerados, com amostra de 261 profissionais de saúde, provenientes de 22 unidades de saúde selecionados por amostragem estratificada, elegidos e alocados aleatoriamente, mediante sorteio, em dois grupos: intervenção (n = 87) e controle (n = 174). Os participantes do grupo intervenção foram expostos a uma capacitação sobre comportamento suicida com duração de 20 horas. Todos os 261 participantes foram avaliados, antes e após a intervenção, e os grupos foram comparados para avaliar a atitude frente ao comportamento suicida utilizando o *Suicide Behavior Attitude Questionnaire* (SBAQ), avaliação feita por comparação das médias via teste t-Student para amostras independentes e Teste t pareado para amostras dependentes.

**RESULTADOS:**

O grupo intervenção, na comparação intragrupos antes e depois da capacitação, bem como na comparação com o grupo controle, demonstrou diferenças estatisticamente significativas nas atitudes frente ao comportamento suicida, avaliadas a partir das diferenças de *scores* nos domínios: “percepção da capacidade profissional”, em todos os quatro itens; “sentimento negativo”, em seis dos sete itens; e no domínio “direito ao suicídio”, três dos cinco itens.

**CONCLUSÃO:**

A capacitação breve desenvolvida na atenção primária à saúde foi efetiva para a melhora das atitudes dos participantes que integravam o grupo intervenção frente ao comportamento suicida.

## INTRODUÇÃO

Aproximadamente 800 mil pessoas cometem suicídio anualmente, o que corresponde a uma taxa de 11,4 mortes a cada 100 mil habitantes em todo o mundo, figurando entre as dez principais causas de morte em todas as faixas etárias^1^. Embora a taxa de mortalidade por suicídio em alguns países da Europa Ocidental tenha diminuído nas últimas décadas, outros países, como México, Estados Unidos e Brasil, experimentaram um aumento dos casos durante o mesmo período^2^. De acordo com dados nacionais, mais de 10 mil brasileiros se suicidaram, ou seja, 5,5 ocorrências a cada 100 mil habitantes em 2015 e em 2016, 6,5 óbitos para 100 mil habitantes, comprovando um crescimento ao longo dos anos^3^. Considerando a magnitude do problema, a OMS recomenda que o suicídio seja priorizado tanto no atendimento oferecido quanto no desenvolvimento de políticas de saúde pública^1^.

Frente a esse contexto, é inevitável que os serviços de saúde sejam acionados, sendo as ações iniciais de cuidados em saúde mental, frequentemente prestadas em ambiente da atenção básica^4^. Tal fato relaciona-se diretamente ao número insuficiente de profissionais na rede de atenção psicossocial para atender a essa demanda, sobretudo em países de média renda, como o Brasil, em que a cobertura de serviços dessa natureza atende somente a 10% dos indivíduos que necessitam^4^.

Uma revisão sistemática analisou o contato dos indivíduos com profissionais de saúde da atenção primária antes de cometer suicídio e os resultados evidenciaram que, em média, 80% procuraram o serviço de saúde no ano anterior ao suicídio, e 44% no mesmo mês do óbito^5^. Achados semelhantes foram descritos em um estudo norueguês, que investigou a utilização dos serviços de saúde da atenção primária em 4.926 casos de suicídio (sujeitos com idade > 15 anos) durante o período de 2007 a 2015. Os resultados comprovaram que aproximadamente 90% dos indivíduos consultaram um profissional generalista na atenção primária no ano anterior ao suicídio e até 46,4% no último mês de vida^6^.

Isso sugere que os profissionais desse nível de atenção estão em uma posição única para identificar sujeitos em risco suicida e intervir, uma vez que a atenção primária à saúde configura-se porta de entrada na rede de saúde^1^. Entretanto, grande parte dos profissionais, frente a pessoas com comportamento suicida, manifestam atitudes negativas^7^, comumente em decorrência de fatores como despreparo ou dificuldades em lidar com essa demanda, proporcionando um atendimento inicial limitado e frequentemente revertido em encaminhamentos protocolares a outros serviços^9,10^, comprometendo a qualidade do cuidado prestado.

Uma atitude pode ser definida como um conjunto de atributos cognitivos, afetivos e comportamentais. Portanto, a atitude é uma inclinação do indivíduo a agir de modo específico em relação a determinadas pessoas, objetos e situações, adquirida socialmente, a partir de experiências pessoais e de fatores de personalidade^11^.

Uma metanálise realizada com pesquisas desenvolvidas até o ano de 2018 apontou atitudes negativas, empatia limitada e algum nível de hostilidade dos profissionais de saúde no atendimento às pessoas com comportamento suicida. Os dados indicaram ainda que treinamento e capacitação sobre como lidar com esses casos promovem atitudes mais positivas^12^. Outros estudos, realizados posteriormente, corroboram esses achados, também em relação à resistência em atender essa clientela; ao cuidado pautado por crenças e estigmas; às atividades técnicas e rotineiras priorizadas em detrimento de suporte psicológico, conhecimento e habilidades deficientes, além da necessidade de treinamento visando facilitar as relações terapêuticas^13^.

Dos estudos que se propõem a analisar os efeitos de estratégias educativas na modificação das atitudes, ainda que escassos na literatura brasileira, mesmo internacionalmente observa-se predomínio de intervenções tendo o ambiente hospitalar como público-alvo^16^.

Diante do exposto, este estudo questiona: as atitudes dos profissionais de saúde da atenção primária poderão ser modificadas após uma capacitação profissional para o atendimento ao comportamento suicida? No intuito de alcançar a resposta para tal indagação, este artigo tem como objetivo avaliar o efeito de uma intervenção educativa sobre comportamento suicida nas atitudes dos profissionais de saúde da atenção primária.

## MÉTODO

Este é um ensaio clínico randomizado controlado, de alocação paralela, com dois braços, inscrito no Registro Brasileiro de Ensaios Clínicos (ReBEC) sob o código RBR-9pmjf5w.

Desenvolvido no município de Cuiabá, no período de maio a agosto de 2017, nas unidades de estratégia de saúde da família (ESF), que se estruturam equipes multiprofissionais, composta por no mínimo um enfermeiro, um médico generalista ou de família, um técnico de enfermagem e agentes comunitários de saúde (ACS), podendo contar ainda com equipe ampliada, comportando profissionais de saúde bucal. O município de Cuiabá-MT possui 70 equipes de ESF, sendo três na zona rural do município e 67 da zona urbana, distribuídas em quatro regionais: 24 unidades na regional norte, 21 na sul, 11 na leste e 11 na regional oeste. Totalizando 298 profissionais de saúde, entre médicos, enfermeiros e técnicos de enfermagem, 697 agentes comunitários de saúde e 86 profissionais de saúde bucal^23^.

A população do estudo consistiu em profissionais de saúde que exerciam a função de médico, enfermeiro e técnico de enfermagem, excluindo do estudo profissionais de equipes localizadas na zona rural, pois compuseram a amostra de teste piloto no presente estudo, além de agentes de saúde e profissionais de saúde bucal (uma vez que o instrumento utilizado não se direciona para tais categorias).

Adotou-se amostragem probabilística por conglomerados e estratificada, sendo a unidade amostral de sorteio a equipe de saúde que é composta por um grupo de profissionais e estratificada por regionais de saúde.

Para a determinação do número de sujeitos, utilizou-se fórmula para dados pareados^24^, onde foram determinadas as médias de mudança de atitude pré e pós-intervenção e desvios padrão amostrais, obtidos a partir de estudo de referência^25^. Foi estimado um desvio padrão de 2,92, considerada uma mínima diferença a ser detectada de 1,0, confiança de 95% e poder de 0,80. Assim, o tamanho inicial da amostra ajustada para população (n = 298) foi de 56 indivíduos. Por se tratar de delineamento amostral por conglomerado, foi estabelecido fator de efeito de desenho em 1,22 e estimada taxa de não resposta em 22%, o que resultou em uma amostra final 87 indivíduos para o grupo intervenção. Para o grupo controle foi considerado 1 para 2, isto é, 174 indivíduos.

Para seleção aleatória dos participantes dos grupos intervenção e controle foi utilizada amostragem por conglomerados e estratificada proporcional ao tamanho da população, considerando como estratos as regiões (norte, sul, leste, oeste) e como unidade de randomização, a equipe de ESF, a fim de obter controle adequado e reduzir viés metodológico. Definiu-se o número de profissionais selecionados em cada região por multiplicação da fração de profissionais pelo tamanho da amostra (87). Posteriormente, para definir o número de equipes a serem sorteadas dividiu-se o número de profissionais de saúde por estrato pelo número de equipes (quatro), totalizando 22 equipes sorteadas aleatoriamente por programa estatístico, conforme ilustra a Tabela 1.

**Tabela 1:** Estratos, total de equipes de ESF, número de profissionais por região, profissionais por equipe, equipes selecionadas e total de profissionais participantes. Cuiabá, MT, 2017.

Estrato	Número de equipes de ESF	Número total de Profissionais	%	Profissionais por equipe	Número de equipes selecionadas	Profissionais selecionados por região
Região Norte	24	115	38,59	4	8	26
Região Sul	21	83	27,85	4	7	19
Região Leste	11	51	17,11	4	4	12
Região Oeste	11	49	16,44	4	4	11
**Total**	**67**	**298**	**100**	**4**	**22**	**68**

ESF: estratégia saúde da família.

Para a identificação da atitude do profissional frente ao comportamento suicida foi utilizado o *Suicide Behavior Attitude Questionnaire* (SBAQ). Composto por situações clínicas frequentemente vivenciadas por profissionais de saúde^26^. Tal instrumento se divide em fatores que são distribuídos em sentimentos que o profissional tem em relação ao paciente com comportamento suicida, a percepção de sua capacidade para o atendimento e o direito ao suicídio; investigando esses fatores é possível medir uma atitude positiva ou negativa. Espera-se então que, de acordo com a média obtida pela soma dos valores em cada questão divididos pelo número total de questões em cada domínio, possamos verificar atitudes mais positivas por meio de pontuações mais elevadas no domínio “Percepção da capacidade profissional” e mais baixas nos domínios “Sentimentos em relação ao paciente” e “Direito ao suicídio”^26^. Para verificar outras variáveis, sociodemográficas (idade, sexo, cor, escolaridade), relacionadas à formação (profissão, especializações, capacitações e/ou formação em saúde mental) e prática profissional (atendimento anterior a pacientes com comportamento suicida), construiu-se instrumento fechado, aplicado com o SBAQ.

No primeiro momento, todas as unidades participantes foram visitadas, a fim de informar sobre o estudo e aplicar os instrumentos. Os profissionais das equipes sorteadas para o grupo intervenção, além de responder os instrumentos (pré-teste), foram convidados a participar da intervenção (capacitação). Os questionários foram previamente codificados e permitiram o pareamento, mas não a identificação pessoal dos participantes. Os participantes ausentes na unidade de saúde na primeira tentativa, foram procurados, mais duas vezes em horários diferentes. Quando foi possível e necessário, as visitas foram agendadas.

Posteriormente, foi oferecida ao grupo intervenção uma capacitação com duração de 20 horas, visando aprimorar a capacidade de reconhecer o grau de risco de um indivíduo com comportamento suicida e intervir; conhecer estratégias de atendimento e/ou encaminhamento de indivíduos com comportamento suicida; reconhecer a própria atitude perante um paciente com comportamento suicida e aperfeiçoá-la. A definição dos conteúdos abordados teve como direcionador o manual de prevenção ao suicídio voltado para equipes de atenção primária da Organização Mundial da Saúde. A capacitação foi conduzida por psicólogas e pesquisadoras em suicidologia, com ampla experiência clínica e pedagógica nessa temática. Imediatamente após o término da capacitação foi reaplicado o SBAQ para o grupo intervenção (pós-teste). Para o grupo controle não foi oferecido nenhum tipo de intervenção e o pós-teste foi reaplicado posteriormente nas unidades de saúde.

O desfecho primário esperado foi diferença no nível de atitudes frente ao comportamento suicida entre o início e fim da intervenção, verificado por meio do SBAQ, a partir da constatação de aumentos de escores igual ou superior a 3%, avaliado antes e depois da intervenção, a análise foi realizada sobre o princípio de intenção de tratar.

A Figura apresenta o fluxograma de participação dos indivíduos envolvidos no ensaio clínico desde o recrutamento até a última avaliação. Dos 87 profissionais iniciais que foram expostos à intervenção, 69 (79,3%) participaram da última avaliação, e no grupo controle permaneceram para análise 88 (50,6%). As perdas do estudo ocorreram por recusa em permanecer no estudo (n do grupo intervenção = 7, n do grupo controle = 11), preenchimento incompleto dos instrumentos (n do grupo intervenção = 11, n do grupo controle = 37), mudança de local de trabalho ou não foram encontrados ( n do grupo controle = 38).

**Figura:** Fluxograma do estudo. Cuiabá, MT, Brasil, 2017.

A distribuição dos dados foi verificada pelo teste de Shapiro-Wilk, para a comparação entre os dois grupos foi utilizado o teste de homogeneidade qui-quadrado de Pearson, teste t-Student para duas amostras independentes e teste U-Mann Whitney. As comparações pré e pós-intervenção intragrupos foram realizadas a partir do teste t pareado para amostras dependentes e teste de postos com sinais de Wilcoxon. O nível de significância adotado foi de 5% em todos os testes. O projeto de pesquisa foi aprovado pelo comitê de ética em pesquisa de acordo com a Resolução 466/2012.

## RESULTADOS

A análise dos dados sociodemográficos, de capacitação anterior e de atendimento ao comportamento suicida (Tabela 2) não evidenciou diferença estatisticamente significante entre os grupos.

**Tabela 2:** Características sociodemográficas, profissionais e de comportamento suicida dos profissionais de saúde das unidades de ESF do município de Cuiabá, MT, Brasil, 2017.

Variáveis	Grupo intervenção	Grupo controle	n (88)	(%)
	n (69)	(%)		
Faixa etária				
20–39	31	44,9	31	35,2
40–59	37	53,6	49	55,7
60–79	1	1,4	8	9,1
Sexo				
Feminino	60	87,0	79	89,8
Masculino	9	13,0	9	10,2
Escolaridade				
Sem curso superior	11	15,9	15	17,0
Com curso superior	58	84,1	73	83,0
Situação conjugal				
Com companheiro (casado, união estável)	36	52,2	57	64,8
Sem companheiro (solteiro, separado, viúvo)	33	47,8	31	35,2
Possui religião				
Sim	58	84,1	79	89,8
Não	11	15,9	9	10,2
Profissão				
Médico	17	24,6	25	28,4
Enfermeiro	18	26,1	24	27,3
Téc. Enfermagem	34	49,3	39	44,3
Capacitação em saúde mental				
Sim	27	39,1	25	28,4
Não	42	60,9	63	71,6
Capacitação sobre suicídio				
Sim	8	11,6	22	25,0
Não	61	88,4	66	75,0
Atendimento a ideação suicida				
Sim	51	73,9	43	48,9
Não	18	26,1	45	51,1
Atendimento a tentativas de suicídio				
Sim	37	53,6	45	51,1
Não	32	46,4	43	48,9
Atendimento a pacientes que se suicidaram				
Sim	18	26,1	20	22,7
Não	51	73,9	68	77,3
Alguma vez na vida você pensou em se matar				
Sim	11	15,9	11	12,5
Não	58	84,1	77	87,5
Alguma vez na vida você tentou se matar				
Sim	2	2,9	9	10,2
Não	67	97,1	79	89,8
Na sua família alguém já tentou se matar				
Sim	27	39,1	20	22,7
Não	42	60,9	68	77,3
Na sua família alguém já se suicidou				
Sim	12	17,4	9	10,2
Não	57	82,6	79	89,8
Entre seus amigos alguém já tentou suicídio				
Sim	27	39,1	21	23,9
Não	42	60,9	67	76,1
Entre seus amigos alguém se suicidou				
Sim	23	33,3	23	26,1
Não	46	66,7	65	73,9

ESF: estratégia saúde da família.

A Tabela 3 descreve as atitudes dos profissionais de saúde do grupo intervenção e do grupo controle, antes e após a intervenção. Os resultados não revelaram diferença significativa, na maioria dos itens do SBAQ, entre os grupos, na medida do pré-teste, exceto pelo item Q17 que apresentou significância estatística com média dos *ranks* (grupo intervenção = 1,40; grupo controle = 2,70; p-valor < 0,001). No entanto, na avaliação realizada após a capacitação, verificaram-se diferenças estatisticamente significativas entre os dois grupos em todos os itens dos fatores “sentimentos negativos em relação ao paciente” e “percepção da capacidade profissional”. Em “direito ao suicídio” dos cinco itens que compõem o fator, os itens Q4, Q6 e Q16 sofreram modificações estatisticamente significativas (p-valor < 0,05), todavia, as questões Q3 e Q18 não apresentaram modificações significativas (p-valor = 0,131 e p-valor = 0,597 respectivamente).

**Tabela 3:** Atitudes dos profissionais de saúde do grupo intervenção e do grupo controle, pré e pós-capacitação. Cuiabá, MT, Brasil, 2017.

Variáveis	Grupo intervenção pré^a^	Grupo controle pré^b^	p-valor^c^	Grupo intervenção pós^d^	Grupo controle pós^e^	p-valor^f^
**Fator 1. Sentimentos em relação ao paciente**						
Quem fica ameaçando, geralmente não se mata.	3,70	4,55	0,020	0,10	4,35	< 0,01
No fundo, prefiro não me envolver muito com pacientes que tentaram o suicídio.	1,00	2,85	0,132	0,10	2,00	< 0,01
Tenho receio de perguntar sobre ideias de suicídio, e acabar induzindo o paciente a isso.	1,70	3,00	0,337	1,00	2,55	< 0,01
No fundo, às vezes dá até raiva, porque tanta gente querendo viver e aquele paciente querendo morrer.	1,70	3,50	0,015	0,40	3,80	< 0,001
A gente se sente impotente diante de uma pessoa que quer se matar.	5,20	5,70	0,524	2,20	5,30	< 0,001
No caso de pacientes que estejam sofrendo muito devido a uma doença física, acho mais aceitável a ideia de suicídio.	1,40	2,70	< 0,01	0,60	2,80	< 0,01
Quem quer se matar mesmo, não fica “tentando” se matar.	1,80	1,85	0,376	0,10	2,00	< 0,01
**Fator 2. Percepção da capacidade profissional**						
Sinto-me capaz de ajudar uma pessoa que tentou se matar.	5,30	5,35	0,914	8,25	5,85	< 0,01
Sinto-me capaz de perceber quando um paciente tem risco de se matar.	4,61	4,31	0,479	8,65	3,70	< 0,01
Acho que tenho preparo profissional para lidar com pacientes com risco de suicídio.	2,50	3,10	0,602	7,50	3,00	< 0,01
Sinto-me inseguro(a) para cuidar de pacientes com risco de suicídio.	5,60	6,15	0,614	3,00	6,45	< 0,01
**Fator 3. Direito ao suicídio**						
Apesar de tudo, penso que uma pessoa tem o direito de se matar.	0,60	1,00	0,618	1,50	1,00	0,131
Diante de um suicídio penso: se alguém tivesse conversado, a pessoa teria encontrado outro caminho.	8,20	8,10	0,934	9,50	8,00	< 0,01
A vida é um dom de Deus e só Ele pode tirar.	9,50	10,00	0,009	8,00	9,50	0,001
Quem tem Deus no coração não vai tentar se matar.	4,60	5,30	0,442	1,60	5,15	0,025
Quando uma pessoa fala em pôr fim à vida, tento tirar aquilo da cabeça dela.	8,90	8,35	0,132	9,00	8,80	0,597

^a^ Grupo intervenção avaliação anterior à intervenção.

^b^ Grupo controle avaliação anterior à intervenção.

^c^ p-valor significativo menor que 0,05 obtido por meio do teste t de Student.

^d^ Grupo intervenção avaliação posterior à intervenção.

^e^ Grupo controle avaliação posterior à intervenção.

^f^ p-valor significativo menor que 0,05 obtido por meio do teste U-Mann Whitney.

Na Tabela 4 observam-se as atitudes dos profissionais de saúde do grupo intervenção, antes e após a capacitação. No fator “sentimentos negativos em relação ao paciente”, observam-se ganhos estatisticamente significativos em seis dos sete itens que compõem o domínio (Q2, Q5, Q9, Q13, Q15 e Q19). Na questão 17, as médias pré-teste, 2,71, e pós-teste, 2,02, (p-valor = 0,070) demonstram diferenças, mas sem significância estatística. No que se refere à “percepção da capacidade profissional” houve modificações estatisticamente significativas em todos os quatro itens desse fator, com p-valor < 0,001. Em “direito ao suicídio”, dos cinco itens que compõem o fator, sofreram modificações estatisticamente significativas os itens Q4, Q6 e Q16 (p-valor < 0,005), as questões Q3 e Q18 não apresentaram significância estatística (p-valor = 0,086 e p-valor = 0,234, respectivamente).

**Tabela 4:** Atitudes dos profissionais de saúde do grupo intervenção, antes e após a capacitação profissional das ESF do município de Cuiabá, MT, Brasil, 2017.

Variáveis	Grupo intervenção Pré^a^	Grupo intervenção Pós^b^	Z^c^	P^d^
**Fator 1. Sentimentos em relação ao paciente**				
Quem fica ameaçando, geralmente não se mata.	3,56	1,12	-5,763	< 0,01
No fundo, prefiro não me envolver muito com pacientes que tentaram o suicídio.	2,13	0,96	-2,778	0,005
Tenho receio de perguntar sobre ideias de suicídio, e acabar induzindo o paciente a isso.	2,90	1,08	-4,244	< 0,01
No fundo, às vezes dá até raiva, porque tanta gente querendo viver e aquele paciente querendo morrer.	3,39	1,26	-4,787	< 0,01
A gente se sente impotente diante de uma pessoa que quer se matar.	5,29	3,06	-4,540	< 0,01
No caso de pacientes que estejam sofrendo muito devido a uma doença física, acho mais aceitável a ideia de suicídio.	2,71	2,02	-1,814	0,070
Quem quer se matar mesmo, não fica “tentando” se matar.	3,12	0,96	-5,462	< 0,01
**Fator 2. Percepção da capacidade profissional**				
Sinto-me capaz de ajudar uma pessoa que tentou se matar.	5,38	8,28	-6,323	< 0,01
Me sinto capaz de perceber quando um paciente tem risco de se matar.	4,61	7,78	-6,138	< 0,01
Acho que tenho preparo profissional para lidar com pacientes com risco de suicídio.	3,53	6,81	-5,606	< 0,01
Sinto-me inseguro(a) para cuidar de pacientes com risco de suicídio.	5,70	3,55	-4,206	< 0,01
**Fator 3. Direito ao suicídio**				
Apesar de tudo, penso que uma pessoa tem o direito de se matar.	1,90	2,58	-1,719	0,086
Diante de um suicídio penso: se alguém tivesse conversado, a pessoa teria encontrado outro caminho.	10,0	7,93	-3,580	< 0,01
A vida é um dom de Deus, e só Ele pode tirar.	8,74	7,04	-4,399	< 0,01
Quem tem Deus no coração, não vai tentar se matar.	4,50	3,25	-3,278	0,001
Quando uma pessoa fala em pôr fim à vida, tento tirar aquilo da cabeça dela.	8,27	9,46	-1,189	0,234

ESF: estratégia saúde da família.

^a^ Grupo intervenção avaliação anterior à capacitação.

^b^ Grupo intervenção avaliação posterior à capacitação.

^c^ Teste de Wilcoxon para amostras pareadas.

^d^ p-valor considerado significativo menor que 0,05.

## DISCUSSÃO

A literatura demonstra que o atendimento ao paciente com comportamento suicida pode ser influenciado por diversos fatores, dentre os quais estão as atitudes dos profissionais de saúde^9,10,12^.

O atendimento ao paciente com comportamento suicida pode acarretar sentimentos de frustração, impotência, culpa, desprezo e raiva^18,27^, além de ser envolto por mitos e crenças, como as que classificam as tentativas de suicídio como formas e ameaças para chamar a atenção e que não irão se concretizar, ou ainda que pessoas com esse comportamento são covardes^9,30,31^. A atitude é passível de modificação, o que pode ser evidenciado no presente estudo; quanto ao “sentimento em relação ao paciente”, após a capacitação, foram observadas mudanças em todos os itens desse domínio. Os achados demonstraram mudanças na compreensão das tentativas e ameaças como potenciais fatores de risco e de que pessoas nessas condições estão em intenso sofrimento, propiciando posturas mais empáticas em relação a esses pacientes.

O fato de o profissional reagir de forma negativa ao atender um paciente com comportamento suicida pode, dentre outros aspectos, relacionar-se com o processo de formação que frequentemente não proporciona ferramentas para o enfrentamento de situações de morte, sobretudo quando associada à escolha do sujeito^31^. O desejo de não viver pode ser conflituoso para os profissionais, uma vez que se subentende que devem salvar vidas. Assim, aspectos como superação dos dilemas de onipotência e onisciência, que costumam causar angústia nessas situações, foram amplamente discutidos na capacitação.

Outro aspecto a ser aventado é o de que o sentimento de ansiedade, por um eventual erro de conduta ou de avaliação, e a ideia de responsabilidade sobre a vida do paciente podem ser expressas pela dificuldade em estabelecer vínculo ou no receio de conversar sobre suicídio com medo de induzir o paciente a cometê-lo^32^. No entanto, as discussões geradas durante a capacitação evidenciaram que os profissionais esquivavam-se menos em se envolver e estabelecer vínculo com pacientes que tentaram suicídio e apresentaram redução do sentimento de impotência. Tal fato pode explicar as mudanças significativas das respostas do questionário nesse fator.

Perceber os próprios sentimentos negativos como mecanismos de defesa, considerar a morte com parte da existência humana e identificar os sentimentos envolvidos no processo de morte e morrer fez parte da metodologia empregada e favoreceu a compreensão e modificação dos sentimentos entre os profissionais. Tais achados corroboram o estudo desenvolvido com profissionais de saúde em que se verificou, após uma capacitação, redução significativa dos sentimentos negativos e melhor precisão na avaliação de risco^19^.

Sobre a “percepção da capacidade profissional”, os achados evidenciaram autopercepção de maior capacidade para o atendimento, melhor preparo profissional e segurança para lidar com pacientes em risco de suicídio. Resultados similares são encontrados em uma intervenção com duração de duas horas, oferecida a partir de um estudo multicêntrico japonês a 74 profissionais de saúde. Observou-se ganhos significativos nas habilidades percebidas, confiança, atitude, maior competência na avaliação do risco suicida e mais confiança ao cuidar de pacientes nessas condições^33^.

Um estudo conduzido na Austrália, com 248 profissionais de saúde participantes de um treinamento sobre comportamento suicida, resultou em aprimoramento do conhecimento, atitudes mais adequadas com consequente ampliação da capacidade de comunicação e confiança em fornecer assistência apropriada^21^. A literatura tem enfatizado que as atitudes negativas com pacientes suicidas entre os profissionais de saúde podem estar mais relacionadas à falta de conhecimento e às incertezas em como cuidar do que com a hostilidade especificamente^20^. Desse modo, a desinformação sobre o suicídio pode perpetuar uma abordagem equivocada.

O conhecimento continuamente atualizado, especialmente no que se refere à avaliação do risco suicida e das opções de tratamento, pode diminuir a ansiedade por falhas e aumentar a percepção da capacidade profissional. À medida que os profissionais compreendem e cumprem suas responsabilidades relacionadas à identificação, avaliação e intervenção terapêutica, na atuação profissional baseada em evidências e no planejamento do monitoramento da pessoa com risco suicida, conscientizam-se de que os fatores relacionados a habilidades e competências profissionais foram contemplados^17^.

Os profissionais de saúde convivem com sofrimento, dor, medo, desesperança, perdas de diversos modos e enfrentam frequentemente processos de morte e morrer. Não é raro o sentimento de impotência e incapacidade diante dessas situações. A crença de que apenas a cura ou a recuperação caracterizam o bom cuidado, enfatizado durante a formação acadêmica e reiterada diariamente pela cultura da obstinação terapêutica, pode vir a contribuir para a insegurança profissional ao se deparar com situações que sinalizem uma possível morte autoprovocada^34^.

Diversos autores destacam ainda a influência de questões de ordem organizacional do serviço, bem como a escassez de estrutura física e principalmente pessoal capacitado, demonstrando as dificuldades e o receio de não ter recursos frente ao inesperado. Durante a capacitação, diversos fatores como os relatados acima foram descritos como limitantes de um atendimento efetivo, acompanhados por sentimentos de insegurança, incapacidade e incompetência. No entanto, foi ressaltado que, ao se reconhecerem os limites e potencialidades, o medo de errar pode ser substituído, gradativamente, pela confiança em intervir^17,21,35^.

O terceiro componente investigado no presente estudo refere-se ao “direito ao suicídio”, em que se verificaram modificações, após a capacitação, em três dos cinco itens que o compõem. No entanto, um dos itens em que não foram observadas mudanças significativas foi a afirmativa: “apesar de tudo, penso que uma pessoa tem o direito de se matar”. Resultado similar foi descrito em pesquisa australiana, após capacitação com duração de um dia: na ocasião apenas 30% concordavam com o direito de cometer suicídio. Segundo os autores, tais achados refletem a natureza dos itens que compõem esse fator (crenças morais e religiosas) profundamente arraigados, com menor expectativa de modificações, mesmo após uma intervenção educativa^21^.

Não cabe aos profissionais de saúde priorizar seus princípios e convicções individuais no atendimento à pessoa com comportamento suicida, com o risco de induzi-lo à adaptação aos padrões sociais, baseados em seus valores e crenças pessoais que podem não ser relevantes para o paciente, pondo em risco o vínculo terapêutico necessário para o atendimento. Assim, o cuidado prestado não pode de forma alguma intensificar o sentimento de culpa na pessoa com ideação ou tentativa de suicídio e em seus familiares^34^.

Quando se questionou sobre a possibilidade de modificar uma decisão de suicídio por meio de uma conversa, perceberam-se efeitos positivos significativos após a capacitação. Porém, na afirmativa “quando uma pessoa fala de pôr fim à vida, tento tirar aquilo da cabeça dela”, que presume o próprio profissional envolvido ativamente no evento, os resultados não foram significativos estatisticamente. Uma possível explicação para o achado refere-se ao fato de que, embora o profissional acredite na possibilidade de prevenção utilizando-se da comunicação terapêutica, ele não se reconhece como um elemento essencial nesse processo.

Lidar com a morte desencadeia inúmeras reações no ser humano, dentre elas a percepção da própria finitude. Atentar para questões exclusivamente técnicas, burocráticas e rotineiras, quando se trata desse tema, faz parte de uma postura de negação da morte, na medida em que fornece poder ao profissional de saúde e ameniza o sentimento de impotência^35^. O foco em cuidar apenas dos aspectos biológicos e o investimento em recursos tecnológicos como alternativas de prolongamento da vida evitam, até certo ponto, não só o contato com a morte, mas também a comunicação terapêutica que poderia dar acesso aos sentimentos do paciente. Assim, indiretamente, os profissionais de saúde evitam o contato com a própria morte e com as próprias emoções^34^.

Deixar de falar ou de pensar na morte conforta, de certo modo, pois alimenta a fantasia de que não concretizá-la em palavras e pensamentos pode, assim, afastá-la. A morte do outro se caracteriza como o anúncio e a antecipação da própria morte, uma ameaça a nós, e o suicídio propriamente dito traduz-se ainda em uma mutilação na sociedade pela quebra do seu curso natural, mexendo em suas bases morais^35^.

O suicídio, por envolver aspectos biológicos, culturais e sociais, demanda que as intervenções educativas compreendam, além do manejo clínico, fatores do sofrimento psíquico envolvido, elementos importantes para a abordagem terapêutica da equipe multidisciplinar. Assim, faz-se necessário repensar estratégias de prevenção ao suicídio que consigam, ao mesmo tempo, fornecer conhecimentos que levem a um olhar menos regulado por julgamentos e regras morais, para que a pessoa que vivencia esse sofrimento seja acolhida em qualquer contexto, possibilitando melhores condições de recuperação e reabilitação social^31^.

Este artigo apresenta como limitação a dificuldade de análises comparativas com outras intervenções, tanto por serem escassas na literatura quanto por diferir substancialmente em relação ao público-alvo, conteúdo ministrado, metodologias de ensino-aprendizagem ou formas de análise dos resultados. Porém, as suas contribuições residem no tipo de delineamento experimental que, por intermédio da conscientização do papel do profissional de saúde e aprimoramento do conhecimento sobre o comportamento suicida, promoveu mudança positiva nas atitudes e possibilitou o aumento da percepção da capacidade e confiança para o atendimento a esses pacientes, conforme sugerem os resultados.

Observaram-se atitudes mais positivas entre os profissionais frente ao comportamento suicida após a intervenção. Tal achado contribui para a qualidade da assistência na atenção primária e reforça a viabilidade de capacitações com vistas a prevenir esse agravo na população. Nesse sentido, os dados evidenciados são relevantes tanto para a produção científica quanto para a realidade dos serviços inseridos no Sistema Único de Saúde.

Os resultados encontrados neste estudo vão ao encontro da literatura existente e colaboram com o panorama científico atual, na medida em que fornecem subsídio para o desenvolvimento de estratégias que contribuam para a redução dos altos índices de tentativas e suicídios no país.

Sugere-se em novos estudos, de avaliação sequencial ou de seguimento, observando se tais mudanças são sustentadas ao longo da linha do tempo; além de verificar a duração dos programas de intervenções educativas, visando otimizar o tempo empregado em tais programações, bem como os conteúdos abordados para validação de material educativo padronizado na intenção de testar e replicar tais resultados em outras populações.
